# P-807. Real-World Use of a Host Response Biomarker Assay in Patients with Suspected Infections in an Urban Emergency Department (ED) – Results of the First 100 Cases

**DOI:** 10.1093/ofid/ofaf695.1015

**Published:** 2026-01-11

**Authors:** Edana Mann, Karen C Carroll, Yu-Hsiang Hsieh, Breana McBryde, Eili Klein, Richard E Rothman

**Affiliations:** Johns Hopkins University School of Medicine, Baltimore, MD; Johns Hopkins University Sch , Baltimore, MD; Johns Hopkins University, Baltimore, MD; Johns Hopkins University School of Medicine, Baltimore, MD; Johns Hopkins School of Medicine, Baltimore, Maryland; John Hopkins University, Baltimore, Maryland

## Abstract

**Background:**

Timely distinction between bacterial and non-bacterial infections in the ED is critical for reducing mortality and improving antibiotic stewardship. In 2024, our ED adopted the MeMed BV (MMBV) assay, which computationally integrates three host immune proteins to generate results within 30-minutes.

**Methods:**

Comprehensive evaluation of the first 100 consecutive patients tested with MMBV, by chart review and expert consensus clinical adjudication to assess adherence to intended use, assay performance, and correlation with antibiotic use. Sensitivity and specificity of MMBV calculated, excluding 14 equivocal results (non-actionable per assay design).

**Results:**

Demographic and clinical characteristics of 100 cases with clinical adjudication and MMBV results are presented in Table 1. Overall, 53% of patients met strict intended indications for use. Immunocompromised (n=20) and no indication of fever (n=15) were 2 main reasons for not met criteria for intended use.

Adjudication classified 36 (36%) bacterial infections and 3 (3%) bacterial-viral co-infections. MMBV categorized 31 and 12 patients as high and moderate likelihood for bacterial infection, respectively. Sensitivity and specificity of MMBV, 89.2% (33/37) and 80.4% (37/46), respectively. No statistically significant differences in diagnostic sensitivity or specificity observed when stratified by strict intended use indication (Table 2). Table 3 summarizes the 5 patients meeting MMBV’s indication for use with discordant result between MMBV and clinical adjudication.

Among those adjudicated with bacterial infection or co-infection, all 39 (100%) received antibiotics in the ED. Among those adjudicated to have non-bacterial infections and categorized as moderate or high likelihood for viral/other infection, 64.9% (24/37) did not receive antibiotics in the ED.

**Conclusion:**

Implementation of the MMBV assay in the ED demonstrated high concordance with clinical adjudication for distinguishing bacterial from non-bacterial infections. In spite of relatively high rates of use outside of strict intended use, consistent performance was observed across indications for use groups. These findings support utility of MMBV as a diagnostic adjunct to guide clinical decision-making and improve ED antibiotic stewardship.

**Disclosures:**

Karen C. Carroll, MD, Abbott, Inc.: Advisor/Consultant|BD Diagnostics, Inc.: Advisor/Consultant|CoDiagnostcs: Advisor/Consultant|Cytovale: Advisor/Consultant|Melio: Advisor/Consultant|Meridian, Inc: Grant/Research Support|Scanogen: Grant/Research Support Yu-Hsiang Hsieh, PhD, AbbVie: Grant/Research Support|Visby Medical: Grant/Research Support Eili Klein, PhD, Beckman-Coulter: Grant/Research Support|Diasorin: Grant/Research Support|MeMed: Grant/Research Support Richard E. Rothman, MD, PhD, Beckman coulter: Grant/Research Support|memed: Grant/Research Support

